# Model for predicting immunotherapy based on M2 macrophage infiltration in TNBC

**DOI:** 10.3389/fimmu.2023.1151800

**Published:** 2023-03-14

**Authors:** Haoming Wu, Jikun Feng, Wenjing Zhong, Xiazi Zouxu, Zhengchong Xiong, Weiling Huang, Chao Zhang, Xi Wang, Jiarong Yi

**Affiliations:** ^1^ Department of Breast Oncology, Sun Yat-sen University Cancer Center, the State Key Laboratory of Oncology in South China, Collaborative Innovation Center for Cancer Medicine, Guangzhou, Guangdong, China; ^2^ The Breast Center, Cancer Hospital of Shantou University Medical College, Guangdong Provincial Key Laboratory of Breast Cancer Diagnosis and Treatment, Shantou, Guangdong, China

**Keywords:** M2 macrophage, immune infiltration, TNBC, immunotherapy, prognosis prediction model

## Abstract

**Introduction:**

Compared to other types of breast cancer, triple-negative breast cancer (TNBC) does not effectively respond to hormone therapy and HER2 targeted therapy, showing a poor prognosis. There are currently a limited number of immunotherapeutic drugs available for TNBC, a field that requires additional development.

**Methods:**

Co-expressing genes with M2 macrophages were analyzed based on the infiltration of M2 macrophages in TNBC and the sequencing data in The Cancer Genome Atlas (TCGA) database. Consequently, the influence of these genes on the prognoses of TNBC patients was analyzed. GO analysis and KEGG analysis were performed for exploring potential signal pathways. Lasso regression analysis was conducted for model construction. The TNBC patients were scored by the model, and patients were divided into high- and low-risk groups. Subsequently, the accuracy of model was further verified using GEO database and patients information from the Cancer Center of Sun Yat-sen University. On this basis, we analyzed the accuracy of prognosis prediction, correlation with immune checkpoint, and immunotherapy drug sensitivity in different groups.

**Results:**

Our findings revealed that OLFML2B, MS4A7, SPARC, POSTN, THY1, and CD300C genes significantly influenced the prognosis of TNBC. Moreover, MS4A7, SPARC, and CD300C were finally determined for model construction, and the model showed good accuracy in prognosis prediction. And 50 immunotherapy drugs with therapeutic significance in different groups were screened, which were assessed possible immunotherapeutics that have potential application and demonstrated the high precision of our prognostic model for predictive analysis.

**Conclusion:**

MS4A7, SPARC, and CD300C, the three main genes used in our prognostic model, offer good precision and clinical application potential. Fifty immune medications were assessed for their ability to predict immunotherapy drugs, providing a novel approach to immunotherapy for TNBC patients and a more reliable foundation for applying drugs in subsequent treatments.

## Introduction

TNBC refers to the breast cancer subtype lacking expression of estrogen receptor (ER), progesterone receptor (PR), and lacking over expression human epidermal growth factor receptor 2(HER2), which account for approximately 15% of breast cancers (1, 2). For decades, chemotherapy has been the main first line therapeutic option for TNBC patients. In the early-stage setting, multiagent chemotherapy is the most commonly given prior to surgery (neoadjuvant therapy) and the tumors show a high response rate with 40–50% of the tumors having pathological complete response (pCR). While the patients whose tumors achieve a pCR have low recurrence rates (e.g. <15% at 10 years), those with residual disease have high recurrence rates (overall about 50% at 10 years). In the metastatic setting, the disease is incurable and again chemotherapy is the main therapeutic option with median OS of 2–3 years (3–5). There have been recent advances that include targeted agents and immunology therapy that have improved the outcomes for TNBC (6). While there are only few targeted agents and immunology therapy admitted to clinical treatment for TNBC, such as antibody drug conjugate Sacituzumab govitecan and anti-PD-1 pembrolizumab (7, 8). The search for more effective therapeutic targets and immunology therapy remains a long way to go.

Macrophages play an essential role in the occurrence and development of TNBC. Tumor-associated macrophages, a necessary component of the tumor immune microenvironment, play a significant role in tumor progression, including driving aggressive cell phenotypes in various cancers (9–11). In solid tumor studies, including breast and lung cancers, TAMS promotes releasing tumor-derived CSF-1 and macrophage-derived EGF by invading tumor cells involving paracrine signaling circulation (12–14). Macrophages are malleable in functions and can change their polarization state from M1 to M2 to adapt to different physiological conditions. Activated M1 macrophages produce type I pro-inflammatory cytokines, participate in antigen presentation, and play an antitumor role. In contrast, activated M2 macrophages produce type II cytokines that promote anti-inflammatory responses and tumor development (15). The activation of MI/M2 polarization can be perfect depending on the unique microenvironments of each tissue and external stimulation (16–19).

This research was conducted to investigate the genes connected with the infiltration of M2 macrophages into TNBC. Following this, a model was constructed for prediction of TNBC prognosis, and patients were classified into high-risk and low-risk categories. This research predicted the therapeutic sensitivity of various immunotherapeutics and created the theoretical foundation for developing novel immunotherapy techniques for TNBC.

## Materials and methods

**Figure 1:**
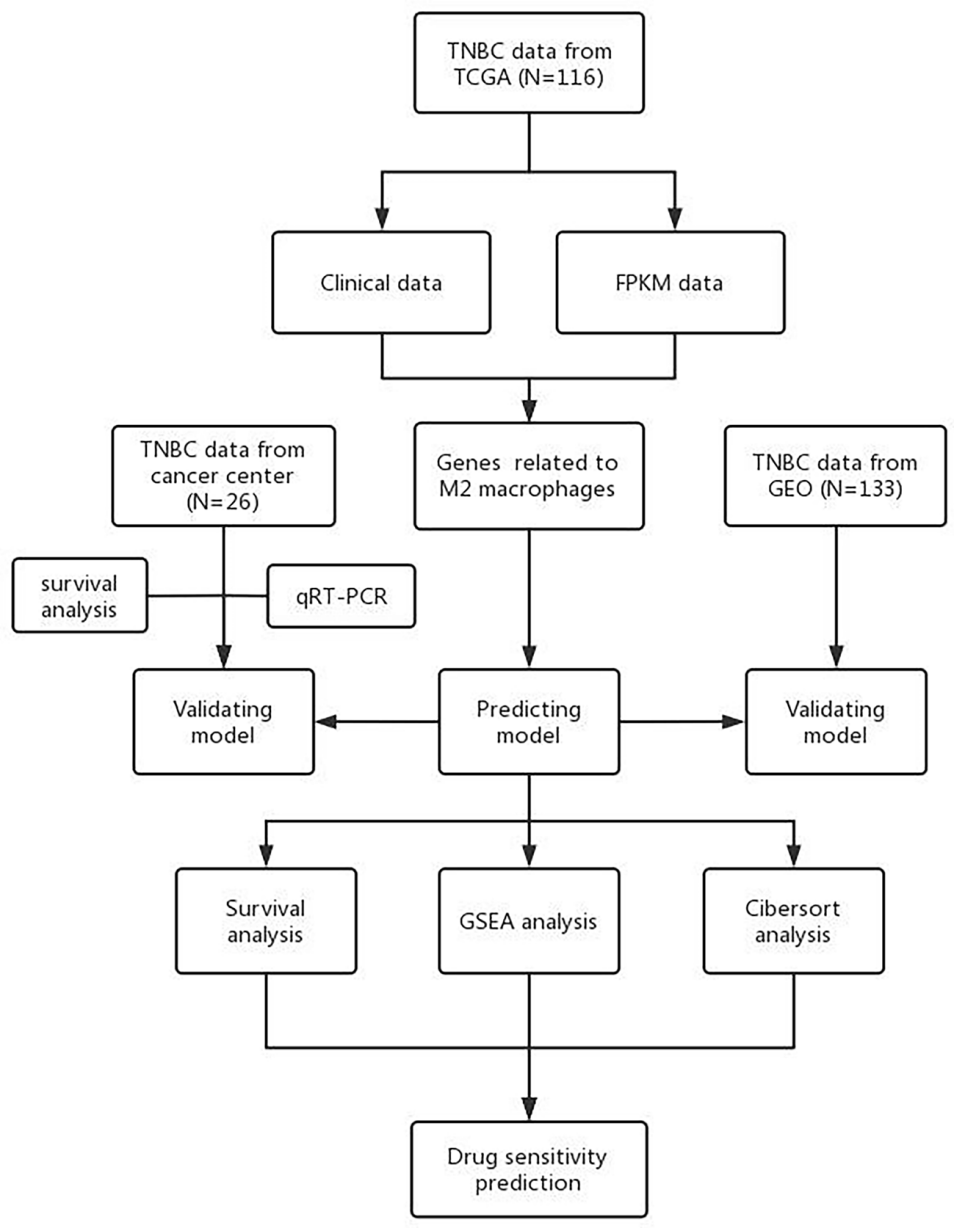
Research design.

**Figure 1** presents research design for this study.

### Gene expression dataset

TNBC dataset was retrieved from The Cancer Genome Atlas (TCGA) and Gene Expression Omnibus (GEO) databases. TCGA dataset comprised basic information, gene expression profiles, and prognostic information retrieved from TCGA database. This study only recruited patients diagnosed with TNBC with confirmed pathological and clinical information. Patients with insufficient or missing data, including age, TNM staging, and OS, were excluded. Information from 116 patients was retrieved. GEO data were retrieved from GEO database by searching keywords: “TNBC” and “survival,” and similar inclusion criteria for TCGA data were used. GSE47994 dataset which contains information from 133 patients, was retrieved from GEO database. Patient characteristics in TCGA and GSE47994 were showed in **Table 1**. And then qRT-PCR was carried out on 26 samples collected from TNBC patient at the Cancer Center of Sun Yat-sen University, and the basic information of patients were showed in **Table 2**.

**Table 1:** Patient characteristics in TCGA and GSE47994 (n = 249 patients).

Variables	No. Patients (%)
Gender	
Female	249 (100.0%)
Male	0 (0.0%)
Age (y)	
≤55	147 (59.0%)
>55	102 (41.0%)
Tumor size	
T1	53 (21.3%)
T2	168 (67.5%)
T3	18 (7.2%)
T4	10 (4.0%)
Nodal status	
N0	113 (45.4%)
N1	74 (29.7%)
N2	43 (17.3%)
N3	19 (7.6%)
Metastasis	
M0	132 (53.0%)
M1	102 (41.0%)
Mx	15 (6.0%)
Pathological grade	
G1	35(14.1%)
G2	73(29.3%)
G3	141(56.6%)
Stage	
I	50(20.1%)
II	79(31.6%)
III	68(27.3%)
IV	102 (41.0%)
Ki-67	
<15%	43(17.3%)
≥15%	206(82.7%)

**Table 2:** Patient characteristics in further validation (n = 26 patients).

Variables	No. Patients (%)
Gender	
Female	26 (100.0%)
Male	0 (0.0%)
Age (y)	
≤55	19 (73.1%)
>55	7 (26.9%)
Tumor size	
T1	2 (7.6%)
T2	18 (69.2%)
T3	2 (7.6%)
T4	4 (15.3%)
Nodal status	
N0	12 (46.2%)
N1	8 (30.8%)
N2	3 (11.5%)
N3	3 (11.5%)
Metastasis	
M0	24 (92.2%)
M1	1 (3.9%)
Mx	1 (3.9%)
Pathological grade	
G1	3(11.5%)
G2	6(23.1%)
G3	17(65.4%)
Stage	
I	2(7.7%)
II	10(38.4%)
III	13(50.0%)
IV	1 (3.9%)
Ki-67	
<15%	6(23.1%)
≥15%	20(76.9%)

### Co-expression analysis

Based on gene expression data in TCGA and information on M2 macrophage infiltration in *CIBERSORTS*, genes related to M2 macrophage infiltration were analyzed using R packages “*limma”* and “*tidyverse*,” where *P* < 0.001 and absolute value of R > 0.45 were set as thresholds. Then R packages “*graph*,” “*reshape2,”* and “*corrplot”* were used to draw co-expression and correlation graphs.

### Signal pathway analysis

Gene Ontology (GO) analysis was performed to explore the biological processes of differentially expressed genes. Kyoto Encyclopedia of Gene and Genome (KEGG) analysis was conducted to identify possible pathways, and results were presented as Sankey plots. GSEA (Gene set enrichment analysis) was conducted based on potential signaling pathways identified for further investigation of their effect in different groups.

### Construction of the prognostic model

Through independent predictive analysis of previously screened genes associated with M2 macrophage infiltration, genes independently associated with TNBC prognosis were determined. This process was implemented using R packages “*survival”* and “*survminer.”* Lasso analysis was conducted based on the selected genes, and cross-validation was performed, where the regression model parameters were finally determined using the point with the minor error (20). This process was implemented using R package “*glmnet.”* GEO data were used to verify regression model accuracy in predicting TNBC patients’ prognoses.

### Risk analysis

According to the regression model, all samples were given risk scores and divided into high- and low-risk groups. Meanwhile, the receiver operating characteristic (ROC) curve was drawn using R package “*timeROC,”* and model accuracy of prognosis prediction was evaluated using the area under the curve. Consequently, the accuracy of risk model in predicting the prognoses of TNBC patients was comprehensively assessed by comparing the four clinical information: age, pathological grade, lymph node metastasis, and clinical stage.

### Further validation for the model

Firstly, the expressions of MS4A7, SPARC and CD300C in normal samples were detected by qRT-PCR, and then calculate the expression mean value and standard deviation (SD). The cut offs were defined as mean +3SD. Then we collected TNBC samples from 26 patients from *Sun Yat-sen University Cancer Center with* all patients’ consent. The following experiments were approved by the Ethics Committee of Sun Yat-Sen University Cancer Center, and the approval number is *GZR-2022-149*. The expressions of MS4A7, SPARC and CD300C in 26 TNBC patients were detected by qRT-PCR, and the TNBC patients were divided into high and low expression group based on the cut-off and results of qRT-PCR. The influence of these genes on the prognosis of TNBC patients was studied by survival analysis. The expression results were then substituted into our model to divide patients into high and low risk groups. The effect of risk grouping on the prognosis of TNBC patients was studied by survival analysis.

## qRT-PCR analysis

Total RNA was extracted from cultured vascular endothelial cells and fibroblasts with Trizol (Invitrogen, Carlsbad, USA). For mRNA detection, cDNA was synthesized from 1 μg of total RNA using the Revert Aid First-Strand cDNA Synthesis Kit (Fermentas, Burlington, Canada). qRT-PCR was then analyzed using the SYBR Premix ExTaqTM II configuration and the ABI PRISM^®^ 7900HT system. The relative standard curve method (2^-ΔΔCT^) used GAPDH as a reference to detect the relative mRNA expression. The PCR primers used in this study are as follows:MS4A7, SPARC, and CD300C

MS4A7-qF: GCTGCGAGAACAGCATCATC
MS4A7-qR: GCCCGTTCTGCAGGTAATCT
SPARC-qF: TTCGGCATCAAGCAGAAGGA
SPARC-qR: GAAACACGAAGGGGAGGGTT
CD300C-qF: CCTCAGGTCCTCCCACGAAG
CD300C-qR: ATTGCTGAACAGGGAGCCAG

### Immunocorrelation analysis

Based on the risk groups, infiltration of different immune cells in different groups, the correlation between them, and correlation of assessed risk with immune checkpoints, including ATIC, OLA1, CTLA4, PDCD1, CD274, IDO1, HAVCR2, and PDCD1LG2, were analyzed. The process was mainly achieved using R packages “*limma”* and “*tidyverse”* combined with *CIBERSORTS*.

### Immunotherapy sensitivity analysis

Combining drug sensitivity file in TCGA database with the risk group data, sensitivity analysis of immunotherapy in different risk groups was conducted using R package “*limma,” “car,” “ridge,” “preprocessCore,” “genefilter,” “sva,” “biomaRt,” “GenomicFeatures,” “maftools,” “stringr,” “Org. Hs. Eg. Db,” “TxDb Hsapiens. UCSC. Hg19. KnownGene,”* and *“oncoPredict”* (21–23). These drugs showing different sensitivity in different groups had therapeutic potential in TNBC.

### Statistical analysis

R software v4.0.3 was used for all statistical analyses. Univariate and multivariate Cox regression analyses were performed to evaluate the survival situation. Hazard ratio (HR) and 95% confidence interval (CI) were calculated to identify genes related to overall survival. Except as otherwise noted, *P* < 0.05 was considered statistically significant.

## Results

### Co-expression results

**Figure 2:**
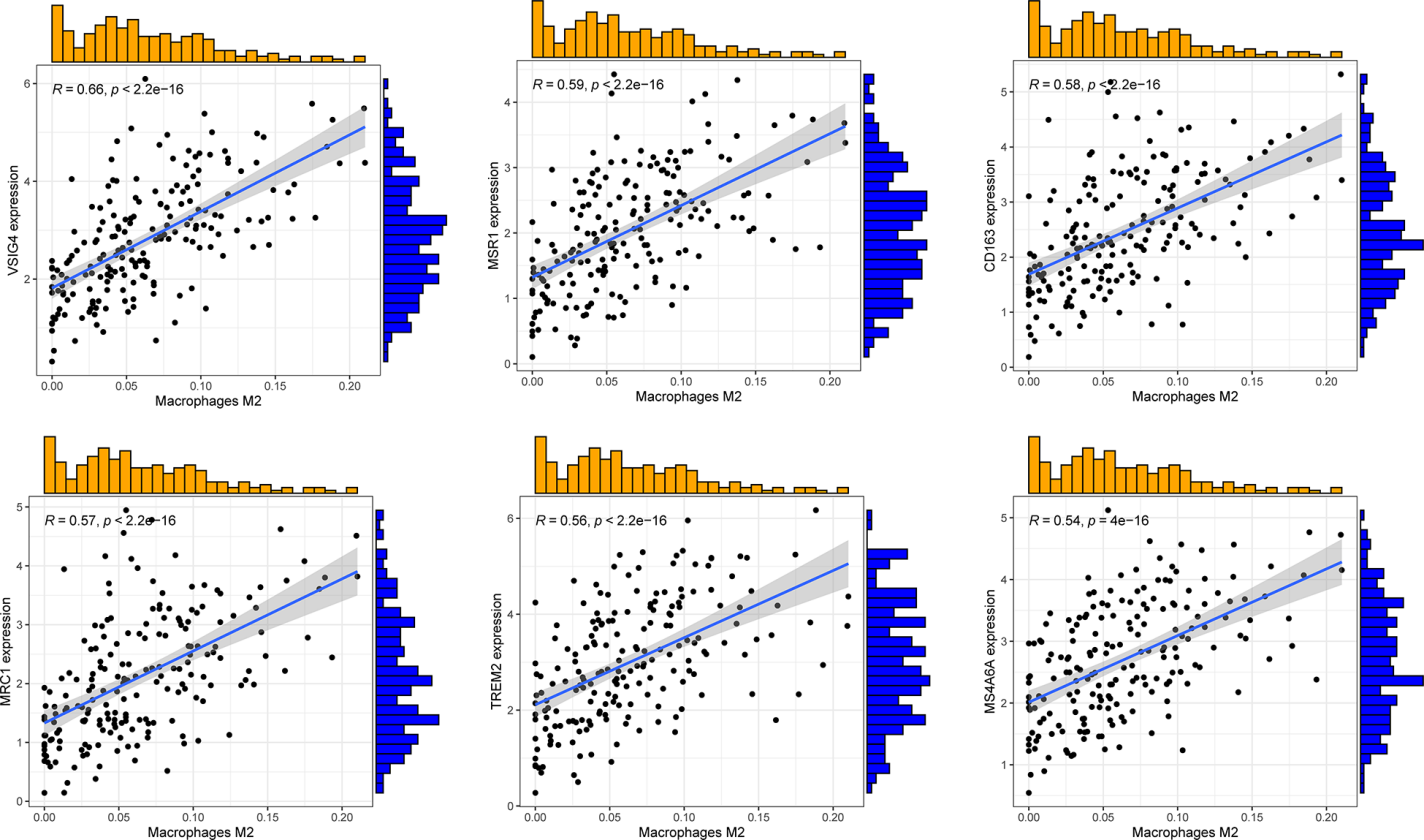
Correlation maps of the top 6 genes associated with M2 macrophage infiltration.

Combining sequencing results of TNBC samples in TCGA database and infiltration of M2 macrophages in CIBERSORT, P < 0.001 and the absolute value of R > 0.45 were set as thresholds. In total, 96 genes related to M2 macrophage infiltration were screened, of which 94 were positively, and two were negatively correlated. **Figure 2** shows the top six genes with the strongest correlation.

### Gene correlation and possible mechanism analysis results

**Figure 3:**
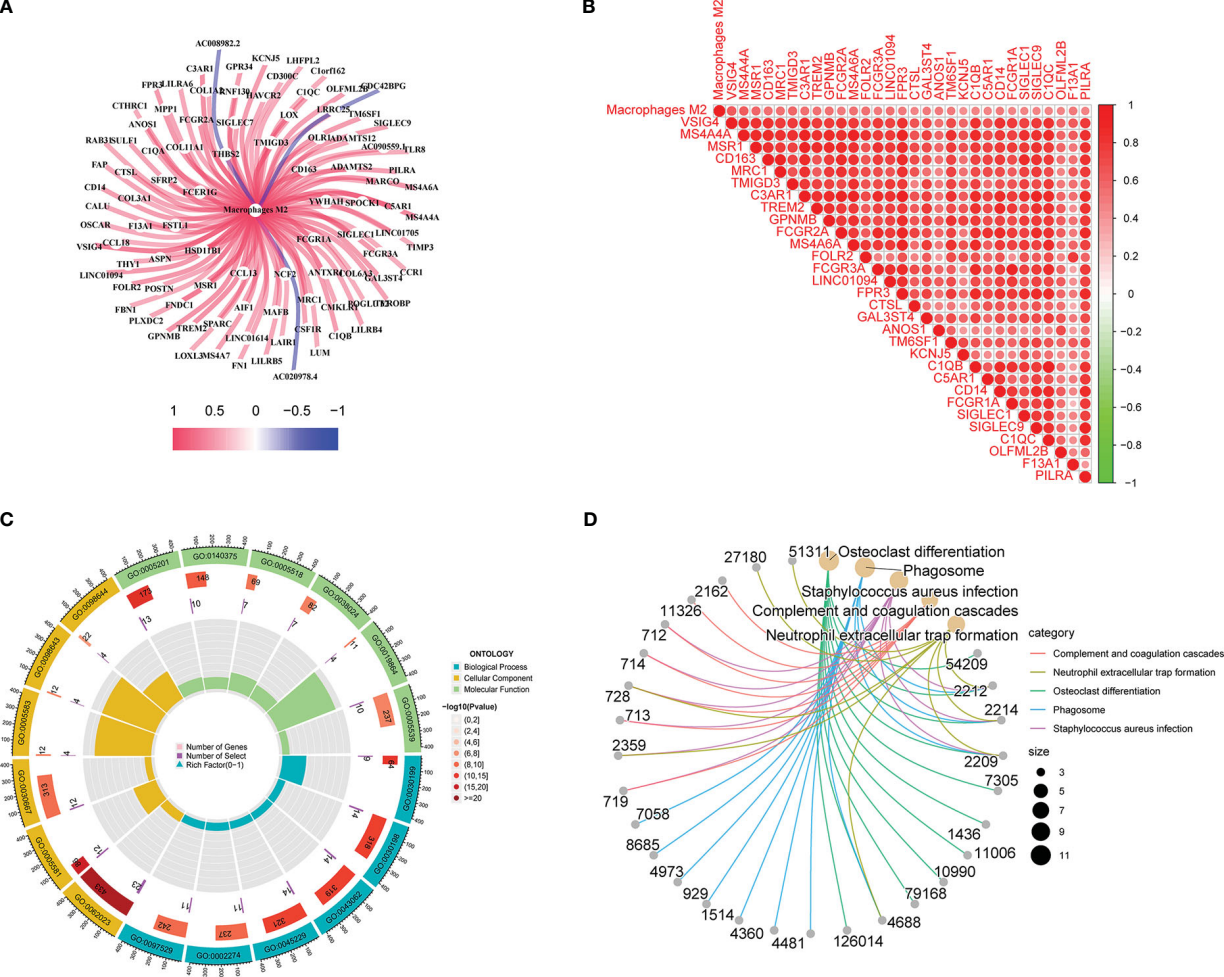
Gene correlation, GO, and KEGG analyses results: **A** shows co-expression network diagram, **B** shows the results of correlation analysis, **C** shows the results of GO analysis, **D** shows the results of KEGG analysis.

Co-expression network diagram was constructed based on the 96 genes screened (**Figure 3A**). Concurrently, a correlation analysis was conducted among genes (**Figure 3B**). Meanwhile, GO, and KEGG analyses were conducted based on these genes to analyze the possible mechanism of their influence on M2 macrophage infiltration (**Figures 3C, D**). In KEGG analysis, phagosome, complement, coagulation cascades, and neutrophil extracellular trap formation were mainly changed. In GO analysis, the extracellular matrix, extracellular structure, and external encapsulating structure organization were the main biological pathway with changes. Collagen−containing extracellular matrix, collagen trimer, and secretory granule membrane were the main cytological component with changes. Immune receptor activity, extractory matrix structural constituent, immune receptor activity, and glycosaminoglycan binding were the main molecular functions with differences. These co-expressed genes influence macrophage M2 infiltration in TNBC through these possible mechanisms.

### Construction and validation of prediction models

**Figure 4:**
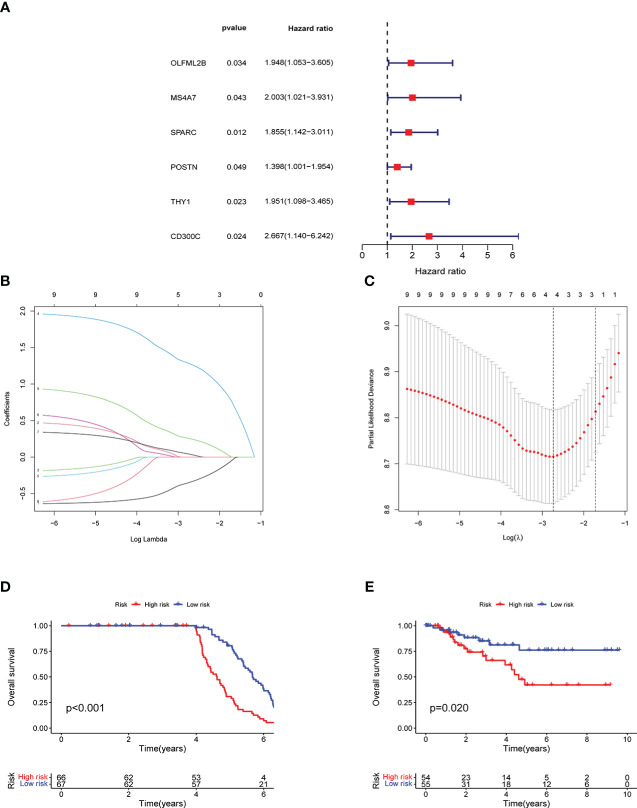
Model construction and verification results: **A** shows the results of univariate prognostic analysis, **B** shows the results of lasso regression analysis, **C** shows the results cross-validation, **D** shows the results of survival analysis in GEO, **E** shows the results of survival analysis in TCGA.

Univariate prognostic analysis was performed on 96 co-expressed genes previously screened, and six genes: OLFML2B, MS4A7, SPARC, POSTN, THY1, and CD300C were screened, which affected the prognoses of patients (**Figure 4A**). Lasso regression analysis was performed on these genes, and cross-validation was carried out simultaneously. Finally, three genes were determined as a reference for model construction, showing the minimum error (**Figures 4B, C**). MS4A7, SPARC, and CD300C were selected as the final scoring genes. Risk scores were performed according to the model on all samples, divided into high- and low-risk groups. Meanwhile, their accuracy was verified in GEO and TCGA data. The prognoses of the high and low-risk groups differed significantly in the two databases (**Figures 4D, E**).

### Validation results by qRT-PCR

**Figure 5:**
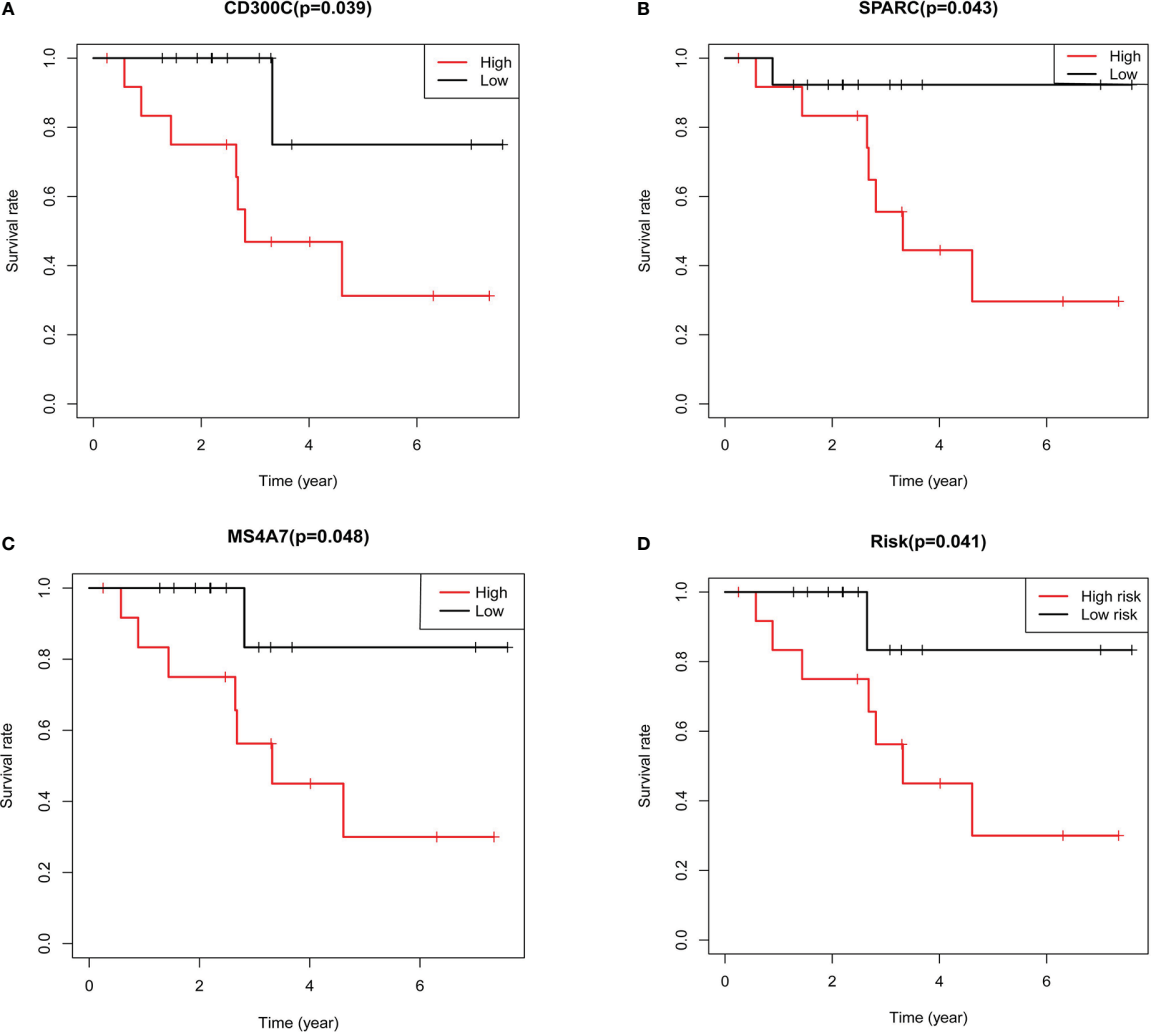
Results of further validation based on qRT-PCR: **A** shows the results of survival analysis based on expression of CD300C, **B** shows the results of survival analysis based on expression of SPARC, **C** shows the results of survival analysis based on expression of MS4A7, **D** shows the results of survival analysis based on the risk grouping.

The different expressions groups of MS4A7, SPARC and CD300C showed significant difference in TNBC patients prognosis (**Figures 5A–C**). Then we plugged the gene expression results in our model, divided TNBC patients into high risk group and low risk group. High risk group and low risk group showed significant difference (**Figures 5D**).

### Analysis of risk scores as independent prognostic factors

**Figure 6:**
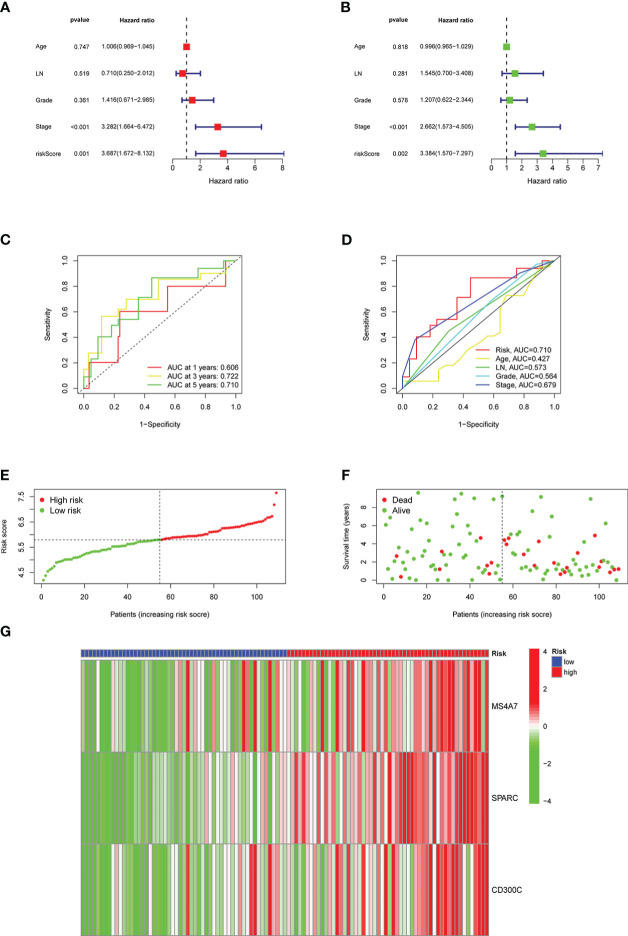
Results of the risk classification and verification analysis as independent factors: **A** shows the results of univariate analysis, **B** shows the results of multivariate analysis, **C** shows the ROC curve using risk score as a single factor, **D** shows the ROC curve using risk score as one of the multiple factors, **E** shows the results of division of high- and low-risk groups, **F** shows the results of survival condition analysis, **G** shows the expression results of MS4A7, SPARC, and CD300C.

In univariate and multivariate analyses, age, lymph node metastasis, pathological grade, clinical stage, and risk score were comprehensively considered. **Figures 6A, B** depict results, and risk scores could be used as independent prognostic factors in both analyses. The ROC curve was plotted using the risk score as a single factor, and the results revealed that the areas under the curve to predict 1-, 3- and 5-year prognoses of patients were 0.606, 0.722, and 0.710, respectively, representing a good prediction precision of risk score (**Figure 6C**). ROC curve was drawn based on multiple factors, including age, lymph node metastasis, pathological grade, clinical stage, and risk score. Our data found that the risk score was significantly better than other factors in predicting the prognosis (**Figure 6D**). **Figure 6E** illustrates the division of high- and low-risk groups. Significantly more patients died in the high- than in the low-risk group (**Figure 6F**). MS4A7, SPARC, and CD300C expressions, the core of prediction model, were significantly different in high- and low-risk groups (**Figure 6G**).

### Predictive effect of risk score on prognosis

**Figure 7:**
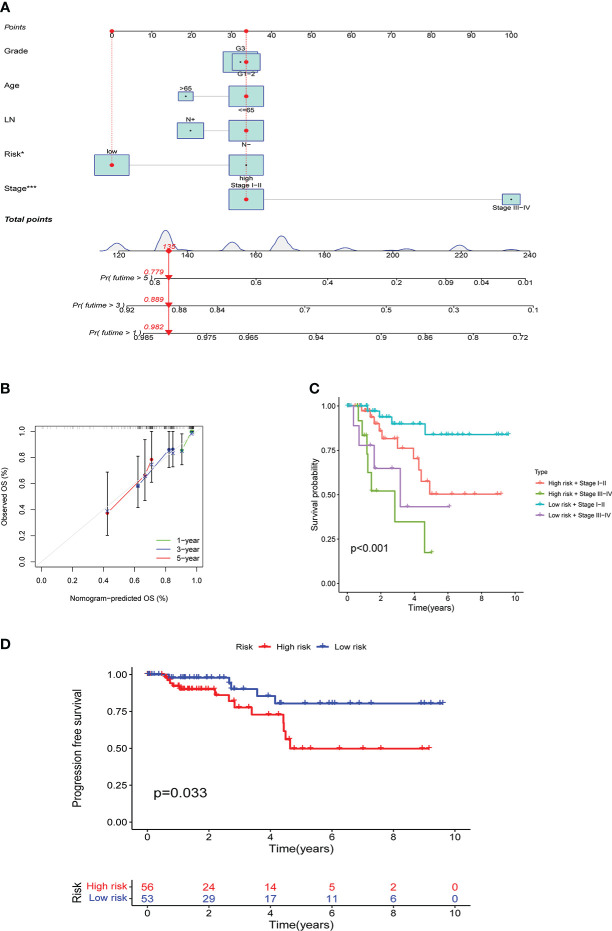
Results of the risk score assessment on the prediction of prognosis of patients: **A** shows the nomogram based on age, lymph node metastasis, pathological grade, risk score, and clinical stage, **B** shows the results of prediction accuracy validation in the model, **C** shows the results of further prognostic analysis, **D** shows the results of PFS analysis.

Based on TCGA data, a nomogram of age, lymph node metastasis, pathological grade, risk score, and clinical stage was drawn, where the clinical stage and risk score were significant to predict the prognoses of patients. Concurrently, predictions of other factors were non-statistically significant (**Figure 7A**). Moreover, our findings revealed that in prognosis prediction based on risk score, the calibrated prediction model for 1-, 3-, and 5-year prognoses were all close to actual prognoses of patients, confirming our risk score accuracy in predicting prognoses of patients (**Figure 7B**). By combining the clinical stages and the risk scores of patients, the further prognostic analysis showed that the high-risk terminal stage group had the worst prognosis. On the contrary, the low-risk early-stage group had the best prognosis, proving that the prognoses of patients could be evaluated more accurately by combining clinical stages and risk scores (**Figure 7C**). PFS analysis was performed in high- and low-risk groups with statistically significant prognostic differences (**Figure 7D**).

### Correlation analysis between risk score and immunity

**Figure 8:**
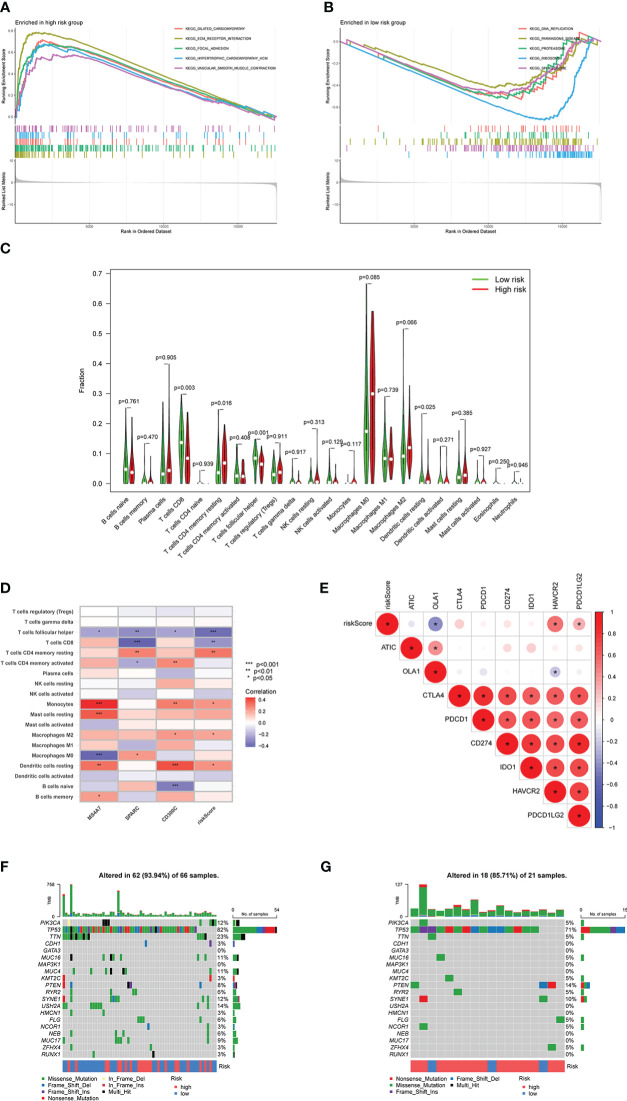
Relationship between risk score and immunity: **A** and **B** show the results of GSEA analysis, **C** shows the infiltration results of immune cells, **D** shows the correlation between MS4A7, SPARC and CD300C with immune cells infiltration, **E** shows correlation between immune checkpoints and risk score, **F** and **G** show the waterfall diagram based on risk scores and tumor mutational burden. * means p<0.05, **means p<0.01, *** means p<0.001.

Based on different risk scores, **Figures 8A, B** illustrate GSEA analysis results and enrichment pathways of the high-risk group, including *KEGG_ECM_RECEPTOR_INTERACTION, KEGG_FOCAL_ADHESION, and KEGG_VASCULAR_SMOOTH_MUSCLE_CONTRACTION*. Enrichment pathways in the low-risk group were *KEGG_DNA_REPLICATION, KEGG_PROTEASOME, KEGG_RIBOSOME*, and *KEGG_SPLICEOSOME*. Simultaneously, CIBERSORT analysis was performed to compare the infiltration of immune cells in the high- and low-risk groups. Infiltration of CD8, CD4 memory resting, follicular helper T cells, and dendritic cells (DCs) resting was statistically significant (**Figure 8C**). Consequently, the correlation between MS4A7, SPARC, and CD300C, the core of prediction model, with immune scores and immune cell infiltration was examined (**Figure 8D**). Follicular helper, CD8, follicular helper, CD4 memory resting T cells, and monocytes were strongly correlated with MS4A7, SPARC, CD300C, and immune score. **Figure 8E** shows a correlation between immune checkpoints and risk score, where OLA1, HAVCR2, and PDCD1LG2 were statistically significant. According to different risk scores and their associated tumor mutational burden, a waterfall diagram was drawn (**Figures 8F, G**).

### Results of immunotherapy drug sensitivity analysis

For these immunotherapy drugs, their association with risk scores was studied. In total, 50 drugs were screened with statistically significantly different drug sensitivity in high- and low-risk groups, which were showed in **Table 3**. KU-55933, Leflunomide, Nutlin-3a (-), and other drugs showed high drug sensitivity and significant differences in different risk groups. All these drugs have good prospects for TNBC immunotherapy. Furthermore, grouping risk scores can help select TNBC patients that are more suitable for these drugs.

**Table 3:** Immunotherapy drug sensitivity and expression differences in different groups.

Drug	Sensitivity of low risk	Sensitivity of high risk	*P* value
AGI-6780	5.8 ± 0.8	6.1 ± 0.9	0.0023
AZD1332	5.6 ± 0.5	5.1 ± 0.2	0.0011
AZD2014	3.7 ± 0.4	3.0 ± 0.5	0.00057
AZD5363	4.7 ± 0.6	4.1 ± 0.3	0.012
AZD6738	2.5 ± 0.2	2.9 ± 0.3	0.0045
AZD7762	0.9 ± 0.2	1.1 ± 0.3	0.039
AZD8055	0.9 ± 0.3	0.8 ± 0.1	0.0053
AZD8186	4.9 ± 0.5	4.5 ± 0.3	0.013
BMS-345541	4.1 ± 0.7	4.9 ± 0.5	0.0014
BMS-754807	1.6 ± 0.3	1.3 ± 0.3	8.7e−05
Cytarabine	2.1 ± 0.5	2.5 ± 0.4	0.015
Dasatinib	2.3 ± 0.6	2.1 ± 0.2	0.001
Dihydrorotenone	1.5 ± 0.2	1.9 ± 0.4	5.2e−05
Doramapimod	6.7 ± 0.8	6.4 ± 0.3	0.0027
Elephantin	4.9 ± 0.2	5, 1 ± 0.4	0.041
Entospletinib	5.5 ± 0.6	5.2 ± 0.4	0.01
Gallibiscoquinazole	3.7 ± 0.3	3.9 ± 0.2	0.0037
GDC0810	7.0 ± 0.3	7.2 ± 0.2	0.014
GSK269962A	4.4 ± 0.3	4.1 ± 0.2	0.00085
Irinotecan	3.2 ± 0.8	3.4 ± 0.6	0.048
JAK_8517	4.8 ± 0.3	4.3 ± 0.2	0.0039
JAK1_8709	6.2 ± 0.3	5.9 ± 0.2	0.036
JQ1	3.7 ± 0.3	3.4 ± 0.4	0.0016
KRAS (G12C) Inhibitor-12	6.0 ± 0.4	6.5 ± 0.3	0.0044
KU-55933	6.55 ± 0.25	6.46 ± 0.20	2.8e−06
Leflunomide	7.1 ± 0.4	7.4 ± 0.4	0.0038
MK-1775	1.3 ± 0.2	1.5 ± 0.1	0.00029
ML323	6.1 ± 0.5	6.5 ± 0.4	0.00012
NU7441	4.09 ± 0.06	3.98 ± 0.03	4.7e−07
Nutlin-3a (-)	7.0 ± 0.5	6.3 ± 0.4	0.016
OF-1	5.7 ± 0.3	6.0 ± 0.2	0.0044
OSI-027	6.61 ± 0.07	6.73 ± 0.05	0.0068
Pevonedistat	1.1 ± 0.5	1.8 ± 0.6	0.0011
PF-4708671	5.8 ± 0.2	5.6 ± 0.3	0.018
PRT062607	4.9 ± 0.4	4.6 ± 0.3	0.038
Ribociclib	5.7 ± 0.1	5.6 ± 0.1	0.012
RO-3306	4.4 ± 0.3	4.2 ± 0.2	0.037
Sabutoclax	0.7 ± 0.2	0.8 ± 0.2	0.011
SB216763	7.6 ± 0.6	7.2 ± 0.8	0.012
SB505124	3.4 ± 0.3	3.2 ± 0.2	0.045
Staurosporine	0.8 ± 0.1	0.6 ± 0.1	0.0033
TAF1_5496	5.3 ± 0.2	5.6 ± 0.3	0.00089
Taselisib	3.7 ± 1.1	3.0 ± 1.0	0.044
Uprosertib	4.8 ± 1.2	4.4 ± 0.9	0.034
VE821	5.7 ± 0.9	6.2 ± 0.7	0.045
Vorinostat	2.2 ± 0.4	2.5 ± 0.3	0.0077
Wee1 Inhibitor	2.6 ± 0.5	3.1 ± 0.4	6.9e−05
WEHI-539	4.7 ± 0.6	5.0 ± 0.4	0.012
Wnt-C59	6.0 ± 0.8	6.3 ± 0.6	0.043
ZM447439	4.4 ± 0.2	4.2 ± 0.1	0.016

## Discussion

In this study, based on the infiltration of M2 macrophages in TNBC and the sequencing data found in the TCGA database, genes that have been shown to have a co-expression relationship with M2 macrophages were investigated. Subsequently, the influence of these genes on the prognoses of TNBC patients was studied. Consequently, six genes were selected, including OLFML2B, MS4A7, SPARC, POSTN, THY1, and CD300C. Genes required for model construction, including MS4A7, SPARC, and CD300C were finally determined using lasso regression analysis, and the model accuracy was further verified using GEO database. The constructed model was used to perform risk scores for patients, and patients were divided into high and low-risk groups. Consequently, the prediction accuracy of model, its association with the immune checkpoint, and the difference in the sensitivity of different immunotherapy drugs were analyzed. Finally, our results confirmed the high accuracy of the prognostic model, and potential immunotherapeutic drugs with the significant application were screened.

Single-cell sequencing findings have confirmed that MS4A7 is highly expressed in some macrophages (24), and can be used as an immune signature to predict ovarian cancer prognoses (25). SPARC, a CR-mimetic adipokine, induces inflammatory interferon response in macrophages during aging (26). SPARC is a tumor suppressor in some studies and a tumor promoter in others, depending on tissue and cell type (27). CD300c is a novel co-inhibitory molecule of T cells that shares a significant sequence homology with existing members of the B7 family. CD300c protein is expressed on professional antigen-presenting cells (APC), including B cells, monocytes, macrophages, and DCs (28). The connection between MS4A7, SPARC, CD300C, and TNBC has received insufficient attention from researchers; as a result, this topic requires additional investigation.

M2 macrophages play a role in promoting tumorigenesis in TNBC by activating several pathways. Genes co-expressed with M2 macrophages were screened, and the correlation of these genes was examined. *Extracellular matrix*, *extracellular structure*, and *external encapsulating structure organization* are some of the biological pathways that are altered in GO analysis, and *collagen-containing extracellular matrix*, *collagen trimer*, and *secretory granule membrane are the main cytological components* that are altered in GO analysis. Additionally, *extracellular matrix structural constituent*, *immune receptor activity*, and *glycosaminoglycan binding* are the primary molecular functions changed in GO analysis. These changes in GO analysis are all possible mechanisms by which M2 macrophages promote tumor development. Moreover, *phagosome complement*, *coagulation cascades*, and *neutrophil extracellular trap formation* in KEGG analysis are also possible mechanisms by which M2 macrophages promote tumor progression. The further researches are necessary for detailed mechanism, which is what we need to do in the future. Furthermore, KEGG analysis revealed that the change in Coronavirus disease (COVID-19) was statistically significant, meaning that COVID-19 infection is very likely to promote M2 macrophages that affect the development of TNBC tumors, which is a novel concept for research.

In the constructed prognosis model, survival analysis of TCGA and GEO data confirmed that the different risk groups had significant differences in prognosis, which was further confirmed using PFS analysis. Also, we revealed that the drawn nomogram could be directly used to calculate the prognoses of patients. Predicted prognoses, whether in 1-, 3-, or 5-year, had little deviation and were consistent with the actual prognoses values. These findings indicate the successful construction of our prognostic model, but more clinical data are still needed for verification.

All types of immune checkpoint medications were examined for high sensitivity and significant variations across risk groups, suggesting they may have a therapeutic effect on TNBC. Using a combined score from three genes, including MS4A7, SPARC, and CD300C, we can identify patients who are unlikely to benefit from immune medication therapy and generate a more precise treatment plan. The fact that our predicted immunotherapy drugs, such as AZD6738, AZD8055, AZD8186, Irinotecan, and Ribociclib, are all successful in treating TNBC, and clinical studies have initiated further demonstrated that our prognosis of immune drugs was entirely accurate (29–33).

In conclusion, MS4A7, SPARC, and CD300C, the three main genes used in our prognostic model, offer good precision and clinical application potential. Fifty immune medications were assessed for their ability to predict immunotherapy drugs, providing a novel approach to immunotherapy for TNBC patients and a more reliable foundation for applying drugs in subsequent treatments.

## Data availability statement

The datasets presented in this study can be found in online repositories. The names of the repository/repositories and accession number(s) can be found within the article/supplementary materials.

## Ethics statement

The studies involving human participants were reviewed and approved by Ethics Committee of Sun Yat-Sen University Cancer Center. The patients/participants provided their written informed consent to participate in this study.

## Author contributions

JY and XW designed the study. HW, WZ and JF finished the main work and wrote the manuscript. XZ, ZX and WH revised and polished the manuscript. XZ and CZ performed the statistical analysis of the data. All authors contributed to the article and approved the submitted version.
